# Social learning about construction behaviour via an artefact

**DOI:** 10.1007/s10071-019-01240-x

**Published:** 2019-02-14

**Authors:** Alexis J. Breen, Clémence C. Bonneaud, Susan D. Healy, Lauren M. Guillette

**Affiliations:** 1 School of Biology, University of St Andrews, Harold Mitchell Building, St Andrews, KY16 9TH UK; 2 Department of Lettres, Sciences Humaines et des Sociétés, Université de Paris, Paris, France; 3 Department of Psychology, University of Alberta, P217 Biological Sciences Building, Edmonton, AB T6G 2R3 Canada

**Keywords:** Animal building, Animal construction, Construction artefacts, Nest construction, Material preference, Social learning, Zebra finch

## Abstract

One source of public information may be the enduring products of others’ behaviour, such as discarded tools or vacated nests. Here, we examined whether observation of a nest affects the material captive zebra finch males prefer when they construct their first nest. It does: for first-time nest construction, males that viewed only an empty cage preferred the colour of material each initially favoured but those males that had observed a pre-built nest of material of their non-preferred colour lost their material-colour preference altogether. Additionally, half of the males that viewed a nest were tested in an environment (the laboratory) different to that in which they were reared (an outdoor aviary). We had expected the aviary-reared (versus laboratory-reared) males would be more uncertain, and thus more likely to select material for their first nest that matched in colour to the colour of the ‘demonstrated’ nest—but this was not the case. The aviary-reared males did, however, tend to touch first the demonstrated colour of material more than did the laboratory-reared males. Together these results show that both observation of a nest and a change in environment can influence the material choices of novice builders. For naïve animal builders, then, construction artefacts can be information resources for learning about potential construction material.

**Electronic supplementary material:**

The online version of this article (10.1007/s10071-019-01240-x) contains supplementary material, which is available to authorized users.

## Introduction

Many animals select raw material from the environment and manipulate it into a species-typical construction, such as a tool for foraging or a nest for sleeping (Hansell 2005; Hansell and Ruxton 2008). These animal constructions, in many cases, can persist long after their use—for example, one-use overnight sleep nests constructed by chimpanzees *Pan troglodytes* can endure for up to 427 days post-occupancy (Stewart et al. 2011). Such animal-made ‘artefacts’ (*sensu* Fragaszy et al. 2013) can inform researchers’ understanding of animal material technologies, for example, by providing historical information, as in the chronometric dating of excavated chimpanzee stone tools, which revealed a 4300-year-old percussive practice (Mercader et al. 2007), or by revealing local material preferences: in a longitudinal study of Corsican blue tit *Cyanistes caeruleus* nests, the materials used by female builders in neighbouring study plots were better explained by the plot rather than the availability of all materials (Mennerat et al. 2009). It is not yet clear, however, whether the animal builders themselves learn anything from the enduring products of others’ behaviour.

There are some data to suggest that animal-made artefacts *may* contribute to learning of construction technique, material selection and/or structural morphology (Fragaszy 2011; Fragaszy et al. 2013). Adolescent tool-using primates and corvids, for example, handle tools that adults discard when foraging (Holzhaider et al. 2010; Humle et al. 2009); chimpanzees seem to select trees for the construction of their night nests based, in part, on the presence of use-wear scars from prior nesting activity that may serve as indicators of a branch’s structural integrity (Stewart et al. 2007, 2011); and New Caledonian crows *Corvus moneduloides* will rip paper into a shape similar in size to a ‘template’ (a large or small paper square—the artefact) that they had learned to drop into a tube to gain access to a food reward (Jelbert et al. 2018). These data, taken together, imply that animal-made artefacts can promote appropriate visual and/or tactile material exploration in a (presumably) appropriate context, with the potential to influence later construction endeavours.

In construction endeavours the choice of material can affect the success of the task. For example, profitable hooked-tool manufacture and use by New Caledonian crows (St Clair et al. 2018) depends largely on the properties of the raw material the crows use: crows that select more rigid plant material construct tools with deeper hooks that, in turn, facilitate faster out-of-reach prey extraction by crows, compared to crow-employed and crafted shallow-hooked tools (Sugasawa et al. 2017). Indeed, deviations in either direction from the optimal (fairly rigid) material can result in crows failing to make hooked stick tools (Klump et al. 2015). The size of materials can also be important. Tree crickets *Oecanthus henryi*, for example, maximize the reach of their mate attraction calls by selecting large rather than small leaves to construct their acoustic baffles (Mhatre et al. 2017). Similarly, breeding male hornyhead chub *Nocomis biguttatus* maximize the resistance of underwater mound nests to high flow rates by selecting pebbles of higher average density (smaller diameter) when compared to non-selected pebbles in the immediate surrounding area (Wisenden et al. 2009). Given the effects of raw-material properties on animal-construction behaviour, it seems plausible that builders might use information provided by construction artefacts to make decisions about material with which to build.

In the current study, we examined whether observational experience of a nest would affect material preference for first-time nest construction by captive zebra finches *Taeniopygia guttata*—a species that readily breeds and constructs nests under laboratory conditions (Breen et al. 2016). In the laboratory, zebra finch males (the builders) prefer to construct their first nest with material of the same colour that they observed a familiar conspecific use for nest construction (Guillette et al. 2016). It may be that the nest itself, however, and not the construction behaviour of the demonstrator bird *per se*, is sufficient to shape a first-time builder’s material-colour preference (Hypothesis I). The ability to learn about potential construction material from the nests of others could reduce unprofitable material-choice decisions by builders, such as choosing visually conspicuous materials (Bailey et al. 2015).

In the wild, zebra finches are opportunistic breeders that will nest in the presence of breeding conspecifics (Mariette and Griffith 2012; Zann 1996). Settlement by zebra finches at a breeding colony is asynchronous, and individuals can arrive at any given point within a breeding period (Griffith et al. 2008; Mariette and Griffith 2012; Zann 1996); with the majority of birds (> 78%) having dispersed from a different natal colony (Zann and Runciman 1994; Zann 1996). The nests of others are, therefore, available for viewing by all but the earliest arriving individuals. Social species such as the zebra finch individuals may be more likely to acquire and use public information if they are uncertain (Boyd and Richerson 1985; Laland 2004), such as when they move into a different environment. The majority of wild vervet monkeys *Chlorocebus aethiops*, for example, that move into a new social group feed first on one of two colours of maize (e.g., pink over blue) that they perceive from observation to be the locally preferred option; the monkeys do this despite migrating from a group where the alternative colour of maize (e.g., blue) was preferred (van de Waal et al. 2013). It seems plausible that individual uncertainty (that is, breeding in a non-natal environment) might also direct zebra finch males’ material-colour preference for first-time nest construction (Hypothesis II).

To test these hypotheses, we manipulated the breeding environment of zebra finch pairs such that half of the pairs bred in their natal environment (because they were reared under laboratory conditions), whereas the other half bred in a non-natal environment (because they were reared in an outdoor aviary). Prior to constructing their first nest, we determined males’ initial preference for one of two colours of material that were tied to a wall of their cage. We then allowed males (and their female partner) to observe, but not interact with, a nest of their non-preferred colour that had been constructed by an unrelated but familiar conspecific. At the end of this observation period we removed the nest and provided pairs with loose pieces of the same two types of coloured material to examine which of these was the preferred option.

## Materials and methods

### Subjects, housing and husbandry

The subjects in this study were bred at the University of St Andrews, U.K. Prior to and after the experiment, all birds were housed in indoor, same-sex free-flight colony rooms, males: 318 × 312 × 230 cm; females: 438 × 251 × 230 cm. Colony rooms were on a 14:10 light:dark cycle, at a temperature of approximately 20 °C, with humidity levels of approximately 50%. Birds were given *ad libitum* access to food (Johnston & Jeff seed, oystershell grit, calcium and vitamin block) and vitamin-supplemented water, in addition to egg mix (up to nutritional independence at ~ 35 days post-hatch; Haith’s egg biscuit food) and spinach three × per week.

### Apparatus

All testing was conducted in one of two test rooms. Each of these test rooms contained two test cages (50 × 100 × 50 cm), which were placed in the centre of the room, back-to-back (10 cm apart), and each contained six perches, two food bowls, two water bowls, oystershell grit, cuttlefish bone and vitamin block at all times during testing. Three 2.4 GHZ Bird Box Cameras (Spy Camera CCTV, Bristol, UK) were fitted to the roof of each test cage (placed at either cage-end or above where we hung the nestbox) to record birds’ behaviour on a laptop computer when testing. See Online Resource 1 for a picture of the apparatus.

## Experiment 1: Experience observing a conspecific nest

### Subjects

Twenty male–female adult (> 90 days post-hatch) zebra finch pairs (i.e., 40 birds) participated in Experiment 1. These pairs had had no experience of (1) viewing or (2) constructing nests or (3) handling construction materials. An additional eight male–female adult pairs (i.e., 16 birds > 90 days post-hatch) constructed demonstrator nests. All pairs that constructed demonstrator nests had previous nest construction and breeding experience (hereafter, experienced birds).

### Demonstrator nests

**Fig. 1:**
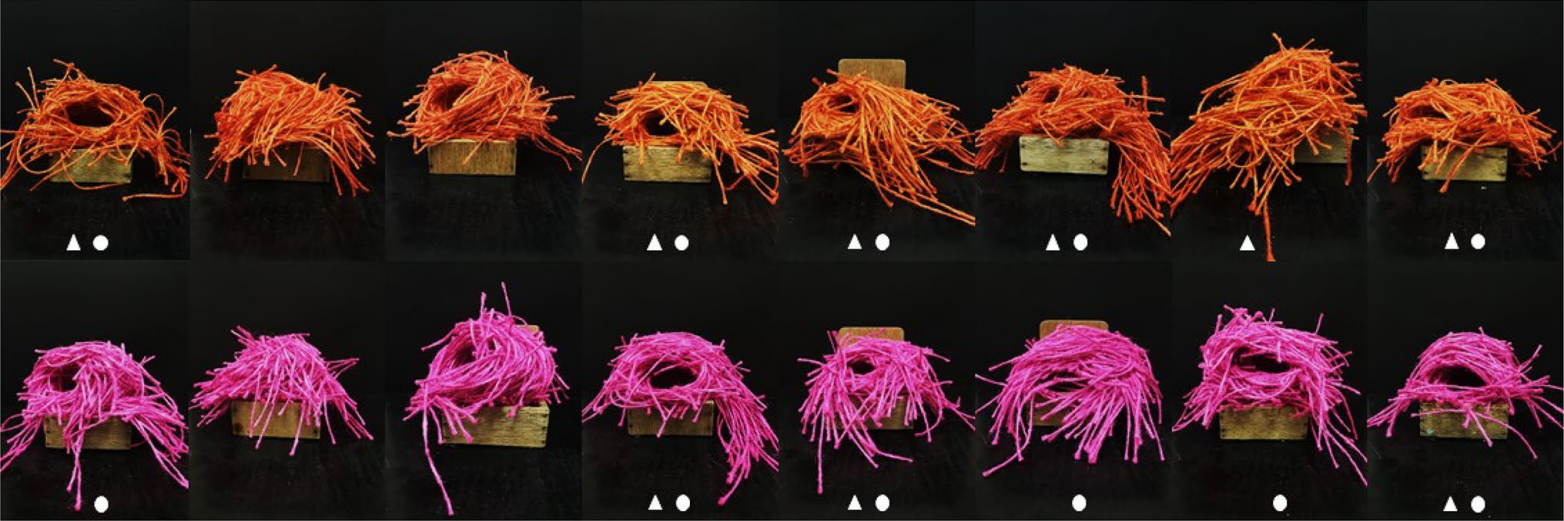
Demonstrator nests. Series of photographs showing the eight orange (top row) and eight pink (bottom row) demonstrator nests, which were available for use in the observation phase in Experiment 1. The geometric symbols represent the nests that were viewed by pairs in each treatment group (aviary, circle; laboratory, triangle). Each column corresponds to the male builder of both coloured nests. All nests were constructed with approximately 400 pieces of 15 cm lengths of string. (Colour figure online)

Eight breeding zebra finch males each contributed two demonstrator nests for Experiment 1: they each constructed one nest with 15 cm long pieces of pink material and a second nest with 15 cm long pieces of orange material (both coloured materials were jute craft twine from James Lever Co., London, UK; Fig. 1). These colours were selected to ensure no colour bias in our experimental design (because adult zebra finch males show no group preference for either of these coloured string types; Guillette et al. 2016). Males were paired with non-related females in cages measuring 50 × 50 × 50 cm and immediately provided with 400 pieces of either pink or orange string and a wooden nestbox (11 × 12 × 4.5 cm) in which to build. Once a male had used all or most of the provided material, we collected the nest and any remaining pieces of material. Pairs were then given a new wooden nestbox and 400 pieces of the other coloured material. Four hundred pieces of material cut to 15 cm lengths is sufficient (from personal observation) for males to construct a species-typical domed nest (Zann 1996), and only nests that were of this shape were used in the current experiment. Males that did not construct a domed nest were allowed a subsequent attempt, up to four weeks, until meeting this criterion. As soon as a male had constructed domed nests of both colours, we returned him and his mate to the group housing conditions described above.

### Experimental protocol

There were two treatment groups in Experiment 1: (1) natal rearing environment, and (2) non-natal rearing environment (hereafter referred to as *laboratory* and *aviary* group, respectively). Birds in the laboratory group (*n* = 10 male–female pairs) were reared in cages (50 × 50 × 50 cm) under the laboratory conditions described above. Birds in the aviary group (*n* = 10 male–female pairs) were reared in an outdoor aviary in St Andrews, U.K. (180 × 240 × 240 cm). Birds reared in the aviary were given the same food, water, supplements, and were monitored by the same animal care staff as birds reared in the laboratory. Because zebra finches do not systematically imprint on the type of material each hatched into (Muth and Healy 2012; Sargent 1965), we did not consider their natal experience of material when choosing experimental subjects. The natal nests of all birds were removed once they (and their siblings) had fledged from that nest (~ 18 days of age; Zann 1996). All birds were housed together as adults in their respective same-sex free-flight colony rooms in the laboratory for seven weeks prior to testing: three weeks for familiarisation with the experienced birds and a subsequent four weeks to allow experienced birds to contribute demonstrator nests (see above). Six days prior to testing we paired males with non-related females from the same treatment group in pairing cages (100 × 50 × 50 cm or 75 × 75 × 40 cm) to allow pair bonds to form. At least sixteen hours before testing commenced, we moved individual pairs into one of the two experimental rooms.

**Fig. 2:**
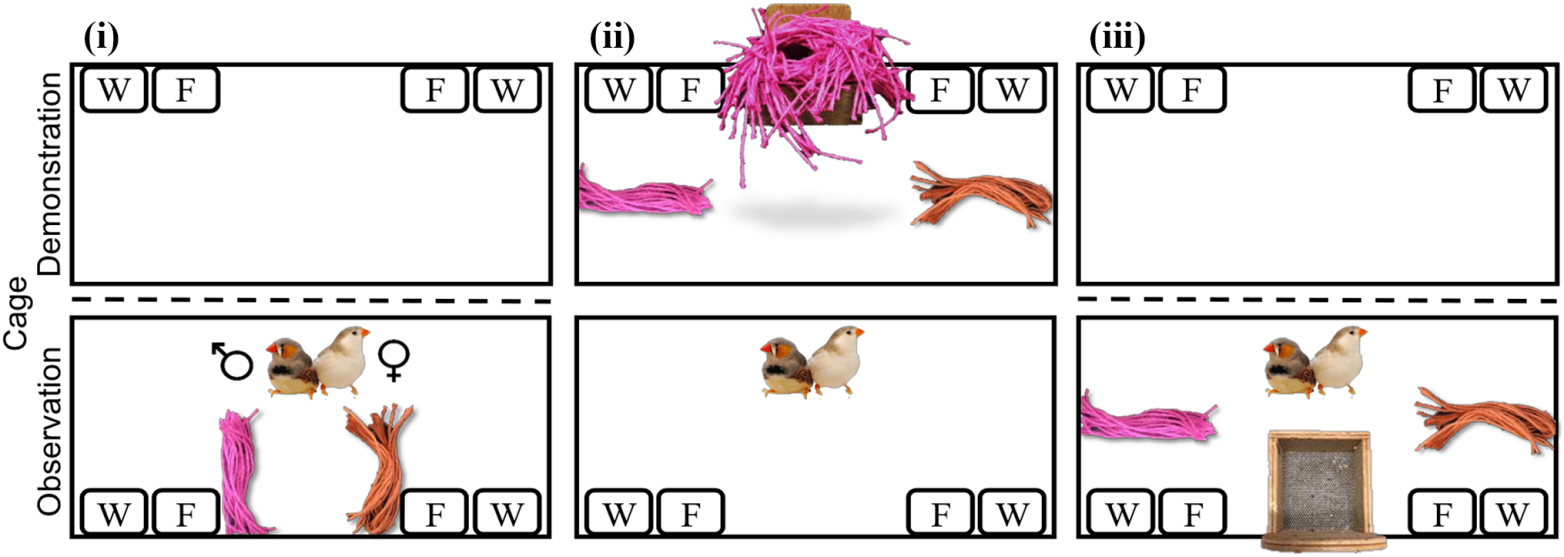
Top-down illustration of Experiment 1 protocol. A male–female pair was housed in one of the two back-to-back facing test cages and the male was first tested for his (i) initial material-colour preference. In this phase the pair was provided two bundles of coloured (pink and orange) material (*n* = 25 pieces in each bundle) cut to 15 cm lengths that were secured to the front cage wall at 40 cm from each cage end. In the subsequent (ii) observation phase, the pair viewed (for 35 daylight h; because the opaque curtain, represented by the dashed line, was removed) a demonstrator nest constructed with material opposite in colour to the male’s initial material-colour preference, and two unsecured 15 cm pink and orange material bundles of the same quantity, in the adjacent test cage midway along each cage wall. In this example, orange was the preferred colour of the observer, so the nest viewed during the observation phase was pink. In the (iii) final material-colour preference test, the curtain divider was returned and the pair was provided a nestbox and 25 pieces of loose pink material and 25 pieces of loose orange material (all 15 cm long). Material position (left or right cage side), with respect to colour, remained consistent across the different experimental phases for each pair—but was randomized across pairs. *W* = water bowl, *F* = food bowl. See Online Resource 1 for an example photograph of the in-cage set-up. (Colour figure online)

Experiment 1 consisted of three test phases (Fig. 2): (i) initial material-colour preference test; (ii) observation; and, (iii) final material-colour preference test. The order in which pairs were tested was randomized within each experimental treatment group.

*(i) Initial material-colour preference test* The initial material-colour preference test commenced one hour after the lights came on the morning after the birds had been placed in the test cage. This experimental phase allowed us to ensure that all males (1) preferred a particular colour of material and (2) did not observe a nest of their preferred colour of material in experimental phase (*ii*). We placed two bundles of coloured material, one pink and one orange, randomized for position across test pairs (left or right cage-side) cut to 15 cm lengths into the pair’s cage, which we then secured by tying each bundle to the front of the cage at a distance of 40 cm from each cage end (Fig. 2i). Each bundle contained 25 pieces of string. The material was secured to ensure that pairs remained naïve with respect to nest construction; they could interact with the material but could not use it to construct a nest. Pairs were allowed to interact with the material for four hours, after which, both bundles were removed from their cage. As soon as this test ended the video recording was scored using Solomon Coder (http://www.solomoncoder.com) version beta 17.03.22 to determine how much time the male spent interacting—the amount of time his bill, feet or body touched the material—with either of the coloured bundles to the nearest 0.2 s. If a male spent at least 30 s interacting with one or both of the material types we considered the material type he interacted with for more time to be his preferred material colour. If, however, he spent less than 30 s interacting with the material we tested his colour preference again the next day (up to four days) using the same procedure described above. The maximum amount of time allowed to complete this initial material-colour preference test was, therefore, 16 h. Throughout this test phase pairs were prevented from viewing the adjacent empty test cage (because we hung an opaque curtain between the two test cages).

*(ii) Observation* This second phase commenced at light onset the day after the initial material-colour preference test. In the demonstrator cage a demonstrator nest that was not of the colour that the observing male preferred (from the initial material-colour preference test) was hung, centrally, from the long cage wall. In addition, two bundles of coloured (pink and orange) material (*n* = 25 pieces in each bundle) were placed on opposite ends of the cage floor (but on the same side as in the previous phase) midway (25 cm) along each cage wall (Fig. 2ii; see Online Resource l for a photograph of the set-up). The use of demonstrator nests was balanced, when possible, both within and across treatment groups for nest colour (e.g., a pair in the aviary group viewed the same pink nest viewed by a laboratory pair) and builder (e.g., pairs within both the aviary and laboratory groups viewed nests of both colours constructed by the same demonstrator bird). After the demonstrator nests and bundles of coloured material were in place, the opaque curtain that prevented pairs from looking into the adjacent demonstrator cage was removed. This experimental set-up, therefore, mimicked the apparent choice of a demonstrator bird that preferred to use only one of the two available materials for nest construction, and the set-up also controlled for any recency effect in the final material-colour preference test described below (because observer males were exposed to both their preferred and non-preferred colours). Pairs were allowed 35 h of daylight (over three natural days) to view the adjacent test cage (and contents), after which, we returned the curtain and the observation phase ended. We chose this time-frame based on previous work (Guillette et al. 2016) that shows male zebra finches will learn about the colour of material, from live demonstration, in 35 h.

*(iii) Final material-colour preference test* The final material-colour preference test commenced immediately after pairs completed the observation phase. Twenty-five pieces of pink material and 25 pieces of orange material (all cut to 15 cm lengths) were placed in the observer pair’s cage in the same position and on the same cage side as described for the observation phase (Fig. 2iii). We then attached a wooden nestbox (11 × 12 × 4.5 cm) inside each pair’s cage midway (50 cm) along the cage front wall (Fig. 2ii). Birds were left undisturbed for the remainder of the day, after which the nestbox was checked every morning and afternoon until the male had placed all of the material of both colours in his nestbox. As soon as the male used all of the material for nest construction, this final test phase ended and birds were returned to same-sex free-flight rooms.

### Behavioural scoring

From the videos the following behaviours by males were scored (see Table 1 for definitions): the colour of material that the males (1) first touched, (2) first picked-up, and (3) deposited into the nestbox (up to the 25th deposit). All scoring was done blind to treatment group using the same behavioural coding software as in the initial material-colour preference test.

**Table 1:** First material interactions. Definitions used for scoring the first material interactions of zebra finch males in the final material-colour preference test of Experiment 1

First material interaction	Definition
Touch	Beak-to or foot-to-material contact
Pick-up	Grasp and lift of material by bill from resting position on ground to elevated position
Deposit	Material brought to and deposited within the nestbox

### Statistical analyses

#### General

All data for this study were analysed in R (R Core Team 2017). We used base R and the ‘lme4’ package (Bates et al. 2015) to run all generalised linear models (GLMs) and the generalised linear mixed model (GLMM), respectively. All GLM(M)s included experimental treatment group (aviary/laboratory) as a fixed effect and were fitted using a binomial distribution and logit-link function; the GLMM included subject ID as a random effect (to account for data non-independence). The goodness-of-fit of all models was confirmed (all *p* > 0.05) using the ‘testUniformity’ function in the ‘DHARMa’ package (Hartig 2017), and Type II likelihood-ratio chi-square tests (from the ‘car’ package; Fox and Weisberg 2011) were used to assess the significance of main effects (Langsrud 2003).

### First material interactions in final material-colour preference test

To determine the effect of breeding environment (natal or non-natal) on males’ first material interactions we specified three GLMs. The dependent variable for all models was whether or not the interaction was directed toward the colour of material of the demonstrated nest: the first touch (yes or no; Model 1); the first pick-up (yes or no; Model 2); and, the first deposit (yes or no; Model 3). We were unable to score the first material deposit of one male in the aviary group because it was the female that deposited all of the material in the nestbox. Thus, for Model 3 (and all analyses below) the final sample sizes were *n* = 9 in the aviary group and *n* = 10 in the laboratory group.

### Final material-colour preference

To determine males’ final material-colour preference, we performed a Monte Carlo simulation to establish that any one of the two colours of material chosen by males > 17 times (out of the first 25 scored) for nest construction differed significantly from chance (*p* < 0.05), i.e., was preferred (Manly 1997). We applied this criterion to each male when we scored his final material-colour preference, as detailed above. Thus, we could classify each male into one of the following two categories: (1) having retained his initial material-colour preference; or, (2) having changed his material-colour preference, either by preferring the colour of material of the demonstrated nest, or by exhibiting no preference for either of the coloured materials after nest observation. Following classification, we specified a GLM (Model 4) to test whether breeding environment (natal or non-natal) influenced whether males retained or changed their material-colour preference after viewing a nest of their non-preferred colour. Next, we specified a GLMM (Model 5) to determine the effect of breeding environment on males’ propensity to use information provided by demonstrator nests about material colour. The dependent variable in Model 5 was the number of material pieces chosen (out of the first 25) in the final material-colour preference test that were of the same colour as the nest each observed. Finally, to test for group-level copying of material that matched in colour to the demonstrator nest, we used one-sample Wilcoxon signed-rank tests with the chance level of 0.5 (no preference) for males in natal and non-natal treatment groups; the dependent variable for both tests was the proportion of pieces (out of 25) each male deposited in his nestbox of the initially preferred colour of material.

## Results: Experiment 1

### Initial material-colour preference

When presented with two bundles of coloured (pink and orange) material, all males interacted with the material for at least 30 s by day four (average amount of time males interacted with the material: 229.52 ± 59.89 s): eleven males (aviary group, *n* = 5; laboratory group, *n* = 6) preferred to interact more with the pink material and nine males (aviary group, *n* = 5; laboratory group, *n* = 4) preferred to interact more with the orange material. As a group, males preferred one of the two coloured materials (average proportion of time males interacted with their preferred material: aviary, 0.76 ± 0.04; laboratory, 0.88 ± 0.04; two-tailed Wilcoxon signed-rank tests: aviary group, *W* = 55, *p* = 0.006; laboratory group, *W* = 55, *p* = 0.002).

### First material interactions in final material-colour preference test

**Fig. 3:**
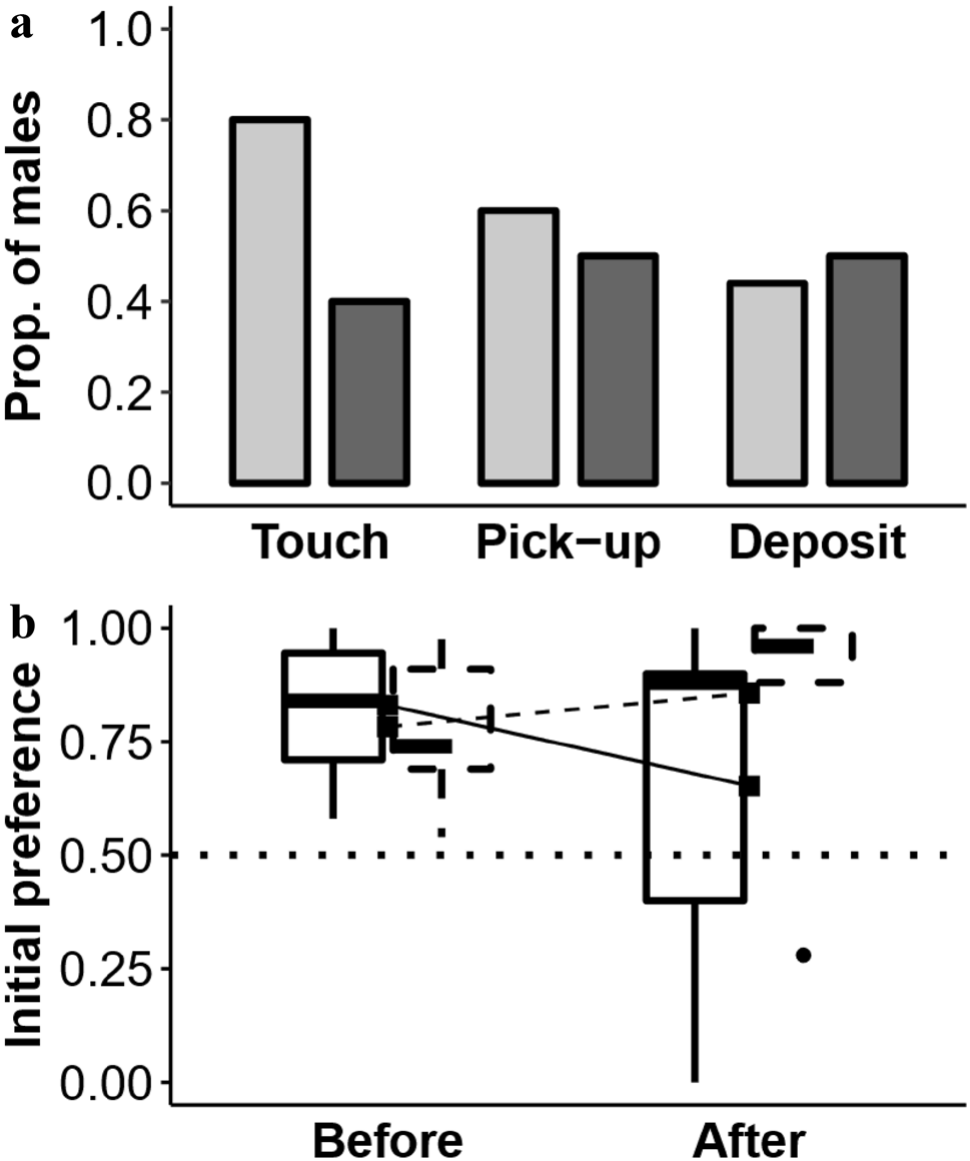
Effect of nest-observation opportunity in the laboratory. **a** The proportion of males (*y*-axis) in the final material-colour preference test that first touched, picked-up, and deposited in their nestbox material of the colour of the nest each observed (*x*-axis). Light grey bars indicate males that bred in a non-natal environment (because they grew up in an outdoor aviary); dark grey bars indicate males that bred in their natal environment (because they grew up in the laboratory). **b** The group material-colour preference by males that did (Experiment 1; solid line) and did not (Experiment 2; dashed line) view a nest for the colour of material each initially preferred (*y*-axis) before and after their observation experience (*x*-axis); boxplots show median values (thick lines), first and third quartiles (lower and upper box quartiles), 1.5 inter-quartile range (whiskers), and an outlier (filled circle); mean values (filled squares) and corresponding trend lines are also shown; the dotted line indicates no preference (chance level)

Aviary males tended to first touch material of the same colour as the nest each had observed, while the laboratory males did not (aviary group, 80%; laboratory group, 40%; Fig. 3a); this effect was marginally significant (GLM: ${\chi ^2}$ = 3.45, *n* = 20, *p* = 0.063; Model 1). The rearing environment, however, had no clear influence (Fig. 3a) on whether males first picked-up the demonstrated material colour (aviary group, 60%; laboratory group, 50%; GLM: ${\chi ^2}$ = 0.20, *n* = 20, *p* = 0.653; Model 2) or deposited this coloured material first (aviary group, 44%; laboratory group, 50%; GLM: ${\chi ^2}$ = 0.06, *n* = 19, *p* = 0.809; Model 3).

**Fig. 4:**
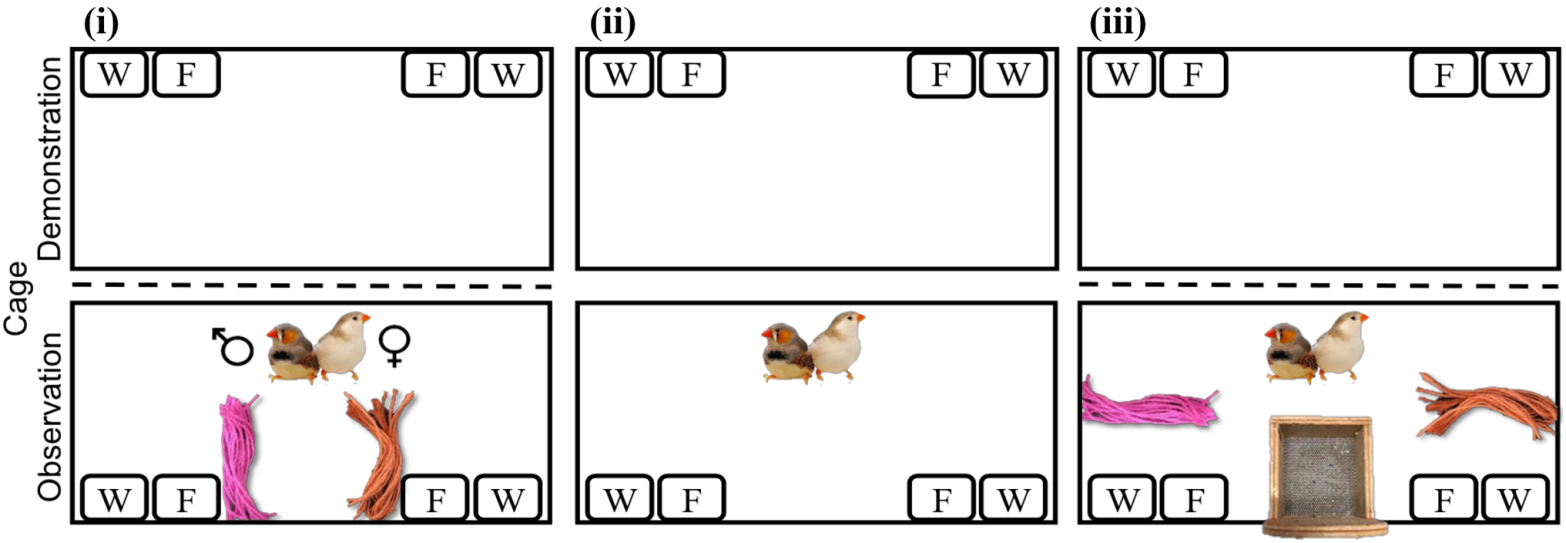
Top-down illustration of Experiment 2 protocol. The three experimental test phases each male–female pair participated in: (i) initial material-colour preference test; (ii) observation; and, (iii) final material-colour preference test, respectively. Experimental protocol as in Fig. 2 except that in observational phase (ii) a male–female pair viewed for 35 h (over three natural days) the empty adjacent test cage (and not a nest or loose material). *W* = water bowl, *F* = food bowl. (Colour figure online)

### Final material-colour preference

Having observed a nest of their non-preferred colour, seven (of 19) males changed their material-colour preference: five preferred to construct their first nest with material of the colour that matched the colour of the nest they had observed and two no longer had a preference. Whether a male changed his material-colour preference after nest observation did not depend on whether he was reared in the aviary or in the laboratory (change/no change: aviary, *n* = 4/*n* = 5; laboratory, *n* = 3/*n* = 7; GLM: ${\chi ^2}$ = 0.43, *n* = 19, *p* = 0.514; Model 4). As a group males did not copy the colour of the demonstrated nest but, after nest observation, their material-colour preference was not different from chance (two-tailed Wilcoxon signed-rank tests: aviary group, *W* = 32, *p* = 0.285; laboratory group, *W* = 35, *p* = 0.472; Fig. 3b). Aviary males were no more likely to choose material that matched the colour of the nest each observed for their first nest than were laboratory males (GLMM: ${\chi ^2}$ = 0.11, *n* = 19, *p* = 0.742; Model 5).

## Discussion: Experiment 1

Males initially preferred either pink or orange material, but after observing a nest of their non-preferred material colour (1) all males’ material-colour preference dropped to chance, and (2) the males breeding in a non-natal (versus natal) environment tended to touch first material of the colour of the nest each had observed, although this apparent between-group difference did not persist as the males began to pick-up and to deposit material.

One interpretation of the first result is that the nest itself affected the males’ material-colour preference when they constructed their first nest. An alternative interpretation, however, is that males’ preference for a particular colour of material decreases with time irrespective of nest observation. This supposition is plausible because when constructing their second nest male zebra finches can prefer, less strongly, their initially favoured colour of material whether or not they built with their preferred material colour or fledged chicks (see Fig. 2 in Muth and Healy 2011).

To determine whether initial material-colour preference in zebra finch males decreases across time, we conducted a follow-up experiment (Experiment 2). In Experiment 2, the protocol was as described for Experiment 1, with zebra finch pairs participating, respectively, in an initial material-colour preference test, observation test phase, and final material-colour preference test, except that pairs were (1) not provided a nest plus material to observe in the observation test phase (and were, therefore, uninformed to what material is ‘appropriate’ for nest construction) and (2) all subjects were reared under laboratory conditions.

If the passage of time (and not nest observation) affects zebra finch males’ material-colour preference for first-time nest construction, males in this second experiment should lose their initial material-colour preference.

## Experiment 2: No nest observation control

### Subjects

Ten male–female adult (i.e., 20 birds > 90 days post-hatch) zebra finch pairs participated in Experiment 2. All pairs were bred under laboratory conditions and were naïve with respect to nest construction.

### Experimental protocol

The protocol for Experiment 2 was just as described for that of Experiment 1, except that in the observation test phase pairs viewed an adjacent test cage that was empty (Fig. 4ii); that is, information concerning nest construction (the apparent material choice and artefact) were unavailable. As the male of one breeding pair did not complete the initial material-colour preference test because he did not interact with the material for ≥ 30 s by the end of day four, we removed this pair from the experiment.

### Behavioural scoring

All videos were scored using the same behavioural analysis software and scoring protocol (here, only part 3) as detailed in Experiment 1. Because two (of the nine) males in Experiment 2 did not construct a nest we could not obtain their final material-colour preference.

### Statistical analysis

The initial and final material-colour preference of males in Experiment 2 were assessed as per Experiment 1. To test for group-level ‘retainment’ of initial material-colour preference in the final material-colour preference test, we used a one-sample Wilcoxon signed-rank test on the proportion of pieces (out of 25) of the initially preferred colour of material each male deposited in his nestbox with the chance level of 0.5 (no preference).

## Results: Experiment 2

### Initial material-colour preference

All but one of the ten males interacted with either or both of the tied-down bundles of pink or orange string for a minimum of 30 s by day four (average interaction time in seconds for the nine males: 383.28 ± 163.55). Of the nine males that remained in the experiment, five preferred pink material and four preferred orange material. Overall, the initial material-colour preference of males, as a group, was significantly different from chance (average proportion of time males interacted with their preferred material: 0.77 ± 0.05; two-tailed Wilcoxon signed-rank test: *W* = 45, *p* = 0.009).

### Final material-colour preference

Together, males that had not had the opportunity to observe a nest preferred above chance level to construct their first nest with the colour of material each had initially preferred (one-tailed Wilcoxon signed-rank test: *W* = 27, *p* = 0.017; Fig. 3b).

## Discussion: Experiment 2

For construction of their first nest, males, as a group, preferred to use the same colour of material as the one they had favoured three days before. It therefore appears that the changes in material-colour preference of the males in Experiment 1 were a result of the opportunity to observe a nest rather than due to the passage of time.

## General discussion

After observing a nest of their non-preferred material colour, zebra finch males had no preference for the colour of material they used to construct their first nest (Experiment 1). Although the material-colour preference of zebra finch males can decrease with time irrespective of prior experience (Muth and Healy 2011), time alone is not sufficient to explain the loss of preference for their favoured material colour by males in Experiment 1, because males that viewed a cage without a nest (Experiment 2) preferred to construct their first nest with the colour of material each initially preferred. The similarity of the environment in which males viewed and constructed a nest to that in which they grew up, however, was unimportant to their material-colour preference. Observational experience of a nest thus affects material-colour preference for first-time nest construction in zebra finch males.

The subtle yet detectable change in males’ material-colour preference following nest demonstration suggests all males *acquired*, but most did not use, the information provided by the nests about ‘appropriately’ coloured construction material. This finding mirrors the behaviour of zebra finch males who watched an unfamiliar conspecific construct a nest with their non-preferred colour of material: these males first interacted with material of the demonstrated colour, but did not use this material when it came to constructing their first nest (Guillette et al. 2016). Together these data show that zebra finch males can be selective in their use of social information that concerns potential construction material. This ability to ‘judge’ others’ nest construction would be adaptive where the nest construction (e.g., choice of site or material) of conspecifics can vary considerably (Zann 1996). It seems likely that males that can identify and avoid adopting ‘bad’ construction behaviour would obtain fitness benefits.

There are at least three possible (non-exclusive) reasons why the effect of viewing a nest was not particularly strong (only five of the 19 males in Experiment 1 of the current study switched their material preference after viewing a nest of the colour they did not prefer). First, it is possible that males reverted to choosing the colour of material they initially preferred after discovering that the demonstrated colour offered no functional advantage, such as inconspicuousness (Bailey et al. 2015) or rigidity (Bailey et al. 2014). Second, it is possible that modifications to the social environment, such as the presence of a seemingly experienced conspecific (e.g., Sherry and Galef 1984), are required to illicit a switch in males’ material-colour preference (Guillette et al. 2016; Guillette and Healy 2018). Third, it is possible that the time frame over which we provided males with information on material ‘suitability’ (colour) was too late in their life history and that, instead, a more appropriate window might be during early adolescence, as is the case for their song learning and sexual imprinting (Zann 1996). Extensive work on the pine cone feeding behaviour of black rats *Rattus rattus* in Israel shows that functionality, social environment, and early learning affect whether animals copy this food processing behaviour (Terkel 1996).

In the absence of opportunity to view a nest, males’ preference for coloured material remained consistent for at least three days. These data give weight to the assertion that observational experience of a nest, and not the passage of time, affects zebra finch males’ material-colour preference. Female zebra finches also continue to prefer the same male of an identical male–male pair in sequential choice tests when there is no intervening experience of a third male but they will decrease preference for their initially preferred male if they are, in an 23-h interim between tests, exposed to a new male suitor who courts them (by singing) frequently (Collins 1995). Thus, it seems that material and mate preference in male and female zebra finches, respectively, are not ‘fickle’ by nature.

Because environmental uncertainty is expected to increase the use of social information, we had expected that the effect of observing a nest under laboratory conditions would be more pronounced in the aviary-hatched than in the laboratory-hatched males (Boyd and Richerson 1985; Laland 2004). We detected a marginally significant trend in the predicted direction: most males (80%) that bred in a non-natal environment touched first material of the same colour as the nest each had observed, whereas fewer males that bred in their natal environment did so (40%). The influence of individual uncertainty on the choices of breeding birds has also been seen in the nest-site preferences of wild migratory flycatchers *Ficedula* species: later arriving females copy the apparent nestbox choice of resident tit *Paridae* species (Seppänen and Forsman 2007). This judgement of whether to copy the locally preferred nesting location does not, however, appear straightforward: timing, prior experience, and the ‘quality’ of the available social information (tit clutch size; Forsman and Seppänen 2011) arguably all play a role. It is conceivable, then, that one or the other or all three of these same factors weighed on zebra finch males’ material-colour preference as, once construction was properly underway, we saw no evident effect of the early environment on later material interactions (picking-up and depositing material into the nestbox).

In conclusion, viewing a nest of a non-preferred colour led zebra finch males to lose their material-colour preference altogether. These data contribute to the growing body of work (Guillette et al. 2016; Guillette and Healy 2017) that shows the circumstances under which zebra finches use information they acquire through experience to be more nuanced than previously thought. It remains to be determined whether other kinds of artefacts, such as animal-made tools, induce similar behavioural changes.

## Electronic supplementary material

Below is the link to the electronic supplementary material.

Supplementary material 1 (DOCX 1136 KB)

Supplementary material 2 (R 5 KB)

Supplementary material 3 (CSV 2 KB)

## Data Availability

Data and R script are available as supplementary material.
