# Supplementary material for: Social learning about construction behaviour via an artefact
Source: Anim Cogn. 2019 Feb 14;22(3):305–15. doi: 10.1007/s10071-019-01240-x (PMC6507502; doi:10.1007/s10071-019-01240-x)

**Breen et al. Supplementary material**

**Online Resource 1. Observational phase in-cage set-up.** Top-down photograph of the experimental test cage layout in phase (*ii*) of Experiment 1, including an example of the material and demonstrator nest presentation.


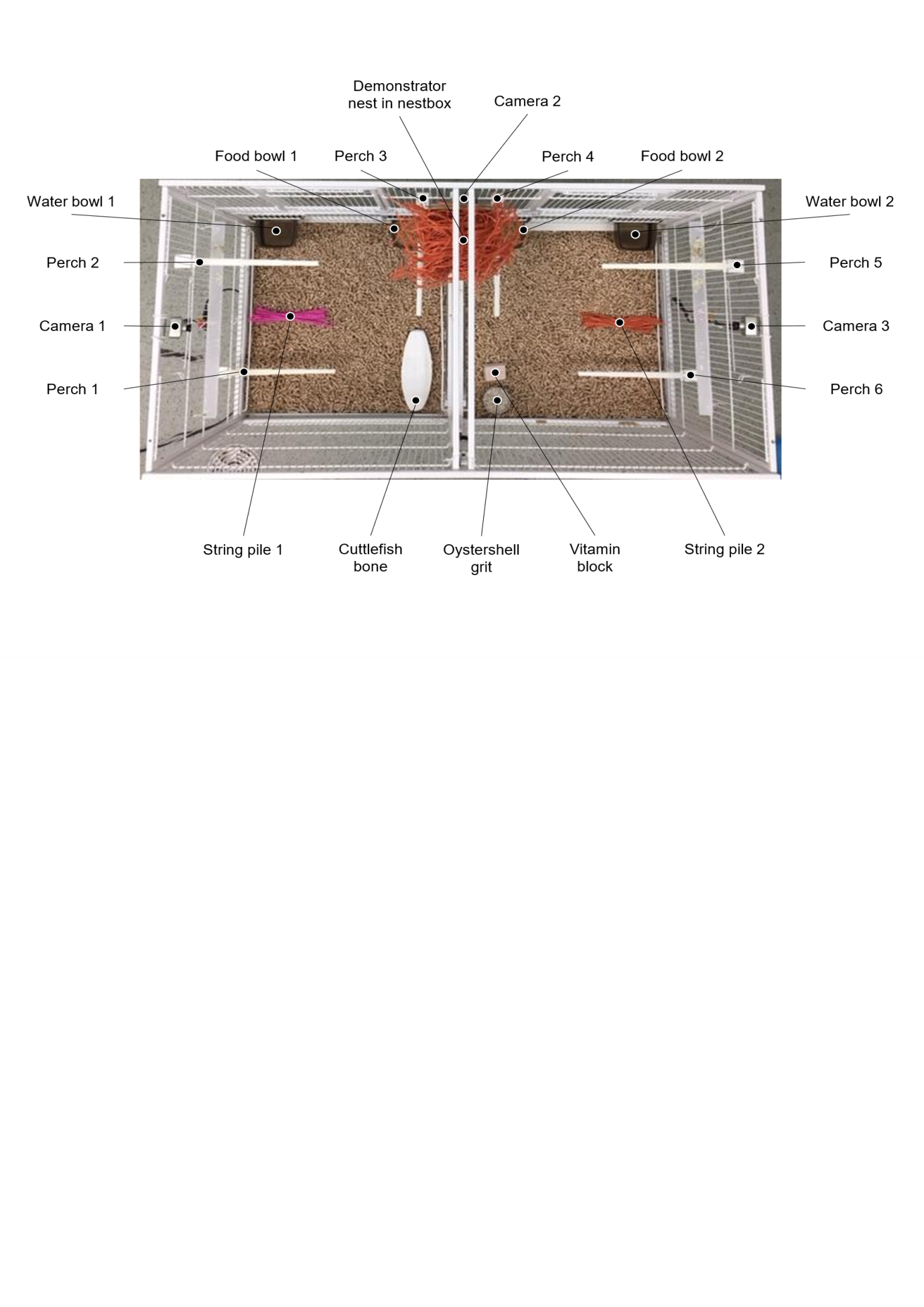

Supplement: Supplementary file 1 — Supplementary material 1 (DOCX 1136 KB) [file 10071_2019_1240_MOESM1_ESM.docx]
